# Insights into early embryonic development - a personal assessment based on 30 years of experience

**DOI:** 10.1590/1984-3143-AR2025-0017

**Published:** 2025-08-04

**Authors:** Urban Besenfelder

**Affiliations:** 1 Institute of Animal Breeding and Genetics, University of Veterinary Medicine, Vienna, Austria

**Keywords:** early embryo development, bovine, endoscopy

## Abstract

The collection of early embryonic stages from the donor animal and their in vitro development through to transfer back into a recipient animal has gained enormous importance both in understanding embryo physiology and in its application for breeding purposes. Whereas not so long ago the focus was on embryo retrieval after superovulation from the donor animal, oocyte collection by follicular puncture followed by in vitro production (IVP) is now the main source of bovine embryos. However, as recent years of intensive research have shown, it appears to be very difficult to reproduce the extreme complex in vivo processes under laboratory conditions. Consequently, the quality/developmental capacity of embryos available for cryopreservation/storage/transport and transfer still lags behind that of embryos derived directly from animals. Embryo collection in bovine MOET programs is limited to the success of the animal's hormone treatment, embryo collection itself, and transfer on Day 7. IVP largely bypasses these developmental steps in the animal and focuses primarily on the presence of healthy follicular waves. It uses follicular puncture (ovum pick-up: OPU) to obtain immature oocytes, which undergo a three-stage in vitro process to produce embryos that are transferred to the uterus on Day 7. However, it is now known that important processes take place in the oviduct that have a lasting effect on the further development of the embryo. In animals, however, the development of embryos in the oviduct has not yet received sufficient attention. This review will present some highlights of the use of early embryonic stages from the oviduct in different species, but the scientific work mentioned is also largely based on the recent presentation at the AETE 2023 conference in Heraklion, Greece.

## Introduction

The use of early embryonic stages in different species plays a central role in the research and development of reproductive biotechnologies. The Fallopian tube fulfills a very important prerequisite in this process, as it is here that the gametes meet in the so-called fertilization process and the resulting embryo undergoes essential developmental steps that optimally prepare it for implantation in the uterus and the further requirements of the fetus. For this reason, over the last three decades our own work has focused on the development and routine use of endoscopy in various species. It is a minimally invasive technique that allows gentle retrieval and transfer of embryos via lumbal or transvaginal access to the peritoneal cavity. It is important to note that, at the same time, precise knowledge is being gained about the response of the ovaries following hormone treatment. Both the information on ovarian response and access to these early embryonic stages are key drivers in bringing the academic pillars closer together with practical application. As I found out during my 10 years as the AETE Secretary, the AETE Award is an extraordinary recognition in an extraordinary scientific community. Being nominated for the AETE Award was so incredible to me that when I received the news, I had to ask for an online conversation with the AETE President - are you sure?

Being selected for the AETE Award and having to write a short overview of my own career made me think and raised some questions: What have you actually done and achieved, when did you start and where was your own fundamental benefit and acceptance to apply in large national and international projects involving numerous colleagues in different countries?

With great respect and recognition for those who have already received the award, I am delighted that Prof. Gottfried Brem is already included in the list of honorees. Professor Brem has been a great mentor and friend since the beginning of my scientific career, and I owe him a great deal. His ideas and suggestions have always been far ahead of their time: he has the extraordinary gift of being able to foresee things far ahead in terms of their importance for science and practice, to help develop them and put them into practice, and thus to promise the greatest possible benefit. It was also he who sent me to my first conference, the AETE conference 1994 in Lyon, where I gave my first presentation on the use of endoscopy in rabbits for reproductive purposes. I did not know the rules of these conferences and, as fate would have it, after my talk on endoscopic oviductal embryo transfer in rabbits, Ray Newcomb stood up and objected that he could not believe that it worked. This was a question from the audience, so I had absolutely no idea how to answer it. That's why my answer was uncertain and very simple: yes, it actually works very well. But Ray Newcomb could tell by the look on my face that he had blown me away with that question. During the coffee break that followed, he told me that he didn't mean to question the technology, but that he thought it was fantastic. This response, in turn, reinforced my belief that endoscopic access to the Fallopian tubes had begun in the rabbit.

As further scientific details are mentioned in detail elsewhere (see also Besenfelder and Havlicek, 2023), this review will only touch on some of the features that describe the use of endoscopy to access the Fallopian tubes in different animal species. Many of the published experimental designs highlighted in this paper were integral parts of our contribution to "Embryo Technology in Europe" as defined by AETE.

## Development of an endoscopic approach in rabbits

During my PhD, I was already working on obtaining embryos from donor rabbits by slaughtering them. Slaughter was the easiest way to obtain a large pool of embryos. All these embryos were surgically transferred into the Fallopian tubes. The recipient rabbits were anaesthetized, clipped around the navel and disinfected, and a 3-4 cm incision was made in the Linea alba, 1 cm behind the umbilical region. The tips of the uterine horns with the corresponding oviducts and ovaries were advanced through the incision site, assessed, and the transfer performed. Each layer of the abdominal wall was sutured individually, the skin was adapted with an intracutaneous suture and the whole procedure was completed with a protective skin fold suture (Besenfelder et al., 1993, 1996). By the time this technique was finally developed into a routine, the procedure took about half an hour to complete. During the following days of wound healing, the recipient rabbits were observed to move little or in unnatural walking patterns. At this point, Professor Brem objected that it was not necessary to use the hands directly to transfer these small embryos and suggested replacing the surgery with a minimal approach using an endoscope. My enthusiasm was limited, especially as I had developed the surgical technique to such an extent that the transfer was quick and easy and no complications were to be expected.

A small skin incision was made with a scalpel (3 mm) near the umbilical area through the subcutis. A rigid arthroscope (4 mm, Hopkins optics, 30° angle, Storz, Tuttlingen, Germany) was selected and introduced through the abdominal wall, muscle layer and peritoneum into the abdominal cavity. This positioning allowed immediate visualization of the entire uterine horns, Fallopian tubes, and ovaries. A venous catheter (13 G, Abbocath-T, ABBOTT, Sligo, Ireland) was inserted through the skin into the abdomen near the infundibulum to the ampulla containing the transfer system, a 1 ml syringe with a 5 µl glass capillary attached (Brandt, Wertheim, Germany). After the transfer was completed, the small skin wound was closed with a Michel clamp.

The laparotomy procedure mentioned above has been described in some detail because the introduction of endoscopy has greatly reduced the material and methodological efforts involved. The time taken to insert the endoscope into the abdominal cavity, transfer the embryo, remove the endoscope and close the wound with a metal clamp was also drastically reduced. On average, the procedure was completed in about 100 seconds. In addition, the rabbits' response to wound closure, including wound healing, was significantly better. When the rabbits awoke from anesthesia, they were able to move without restriction and locomotion was judged to be normal. All 30 rabbits in which the endoscopic transfer technique was used for the first time became pregnant. Measured by the implantation rate, routine endoscopic use led to an approximate doubling (Besenfelder and Brem, 1993; Besenfelder et al., 2000).

After the development of endoscopic embryo transfer, all reproductive procedures at the Institute of Molecular Animal Breeding at the LMU Munich, and later at the Institute of Animal Breeding and Genetics at the University of Veterinary Medicine Vienna, were performed endoscopically rather than surgically. Studies comparing repeated embryo transfer in recipient rabbits with artificial insemination showed that embryo transfer resulted in equal or even higher pregnancy rates and that the repeated use of the animals for transfer had no negative effect on reproduction (Besenfelder et al., 2010).

Most embryos were obtained from the oviducts of donor rabbits at the zygote stage at slaughter. Following the successful development of the oviduct transfer technique, embryos were also flushed from the oviduct. Individual rabbits of a particular breed or known genetic background could then be successfully and repeatedly endoscopically flushed (Besenfelder et al., 1998a). To date, more than 10,000 rabbit embryos have been transferred in this way for various purposes such as cryobanking, generation of transgenic rabbit lines for biomedical purposes and cloning (Bolet et al., 2000; Grosse-Hovest et al., 2004; Aigner et al., 2000; Nowshari et al., 2002).

After the use of endoscopy for collection and transfer in rabbits proved very successful, this application was transferred to some livestock species.

## Establishment of endoscopy in reproduction in different species

In small ruminants, particularly sheep, the use of laparoscopy to diagnose pregnancy and to study ovarian processes such as ovulation and follicular dynamics has a long tradition (Snyder and Dukelow, 1974). In addition, it was soon recognized that environmental, semen and female influences could be optimized through breeding hygiene measures (Shackell et al., 1990; Ehling et al., 2003; Kühholzer et al., 1997b; Anel et al., 2006). It quickly became apparent that this technique was also particularly useful for the surgical collection of embryos from donor animals as part of laparoscopic insemination for superovulation treatment (Forcada et al., 2012; Lymberopoulos et al., 2001).

Own work focused on different ways of obtaining embryos from sheep and goats. One method used ovarian follicular dynamics to obtain oocytes from animals using OPU. Donor animals with and without hormone stimulation, in season and out of season and, and donors with reproductive problems could be used to obtain oocytes for IVP in a minimally invasive manner via endoscopically controlled OPU (Stangl et al., 1999; Kühholzer et al., 1997a).

The endoscopic approach was chosen as the next step to transfer embryos into the oviduct of sheep and goats. With the anaesthetized recipients in a dorsal recumbent position, a trocar for the endoscope and a trocar for the forceps were placed 2-3 cm anterior to the udder. A glass capillary was filled with embryos and a venous catheter (13G, Abbocath-T, ABBOTT, Sligo, Ireland) was used to introduce the capillary transcutaneously through the abdominal wall via the Infundibulum into the Ampulla. It has been successfully demonstrated in both sheep and goats that the transfer of early embryonic stages leads to pregnancy (Besenfelder et al., 1994; Kühholzer et al., 1998b). By additionally inserting an embryo flushing catheter into the uterine horns, embryos could also be retrieved from the Fallopian tubes via the uterus after superovulation treatment and endoscopic insemination (Lymberopoulos et al., 2001; Kühholzer et al., 1998a). These methods then became the basis for establishing or maintaining breeding hygiene in flocks, e.g. to achieve virus free herds or to use small ruminants for biomedical models (Vainas et al., 2006).

These developments in small ruminants also paved the way for the endoscopic use of the oviduct in pigs. For a long time, the collection and transfer of embryos in pigs was limited to the surgical approaches (Cameron et al., 1989). Over time, however, other methods have been developed to obtain embryos from the uterus or transfer them to the uterus non-surgically or minimally invasively (see Hazeleger and Kemp, 1999; Rátky and Brüssow, 1995; Brüssow et al., 2000).

Own work built on these successes and focused on the collection and transfer of oviduct stage embryos in pigs. Anaesthetized animals were restrained in the dorsal recumbent position and 3 trocars were placed in the caudal abdominal region in the form of a triangle to accommodate the endoscope, grasping forceps and transfer system. For flushing, an embryo flushing catheter was inserted into the uterine horns instead of the transfer system and held in place with additional grasping forceps. Tubal flushing was performed in two steps: First, one side was flushed in 7 animals, and then both sides were flushed in a further 10 animals. It was impressively demonstrated that this flushing procedure was comparable to embryo recovery after slaughter. Interestingly, two of the unilaterally flushed animals were not slaughtered after collection and gave birth to 6 and 9 piglets respectively. In this method, embryos were subsequently transferred into the oviduct using a glass capillary and more developed embryos were also transferred transmural to the uterus (Besenfelder et al., 1997). In a further optimization, embryos were transferred via a flexible venous catheter (Ø 1.4 mm, 50 cm, with X-ray strip, B. Braun, Melsungen, Germany). The catheter was loaded with embryos under the microscope and then advanced deep into the oviduct (8-10 cm) under endoscopic control. Three recipient animals each received 16 embryos transferred unilaterally. Implantations were determined after slaughter and 8, 16 and 16 implantations were determined. Endoscopic oviductal transfer resulted in a pregnancy rate of approximately 90% in routine transfer programs (Besenfelder et al., 1998b). In conclusion, the use of endoscopy for embryo transfer in pigs offers significant advantages, including minimally invasive application and improved efficiency as reflected in higher pregnancy rates.

## Study of early embryonic stages in cattle

### Technical approach

Finally, all the experience and expertise gained from applications in rabbits, sheep, goats and pigs was projected and applied to cattle. This application in cattle was preceded by the development of laparoscopy to assess ovarian findings for follicular puncture and oocyte collection (Laurincík et al., 1991) and the first attempts at successful flank embryo transfer (Fayrer-Hosken et al., 1989). On the one hand, the advantages of laparoscopy were already apparent, but on the other hand it became clear that percutaneous access to the abdominal cavity from the flank or from the midventral position still required a further reduction in invasiveness and thus stress for the animal. The work of Reichenbach et al. (1993) which described a transvaginal approach to follicle aspiration, was the method of choice.

### Instruments

For the transfer of embryos to the Fallopian tubes and for flushing of the oviductal stages, transfer capillaries or metal flushing capillaries were specially manufactured for this purpose, that could be inserted through the endoscopic tubes into the peritoneal cavity and from there into the Fallopian tube. A 10 µl glass capillary from Brand (Wertheim, Germany) was used as the transfer capillary (see Havlicek et al., 2018). The capillary was rounded at both ends over the Bunsen burner. In addition, one end was bent over the burner flame so that the capillary took the shape of a question mark. This type of curvature allowed the tip of the capillary to thread into the oviduct after it had been inserted through the infundibulum into the ampulla by pushing it forward. In this way, the embryos could be placed 6-8 cm into the tube without the opening of the capillary damaging the epithelium, e.g. by desquamation. An important point here was that the forward exposed opening of the capillary allowed the embryos to flow deeper into the Fallopian tube. It was important to prevent the embryos from flowing back along the inserted capillary. The glass capillary was connected to a perfusion tube with a 1 ml syringe. An embryo transfer gun (IVFETFLEX, STEINER, Austria) allowed precise control of the fluid volume for embryo transfer.

A metal capillary was designed and manufactured (Josef Babicky GmbH, Vienna, Austria) to match the size and shape of the glass capillary. In addition, an olive-shaped bulge was attached to the front end, which could be palpated after insertion and manually fixed in the Fallopian tube, thus serving as a check for correct position. The flushing capillary was also attached to a perfusion tube and connected to a 3-way valve (Braun, Melsungen, Germany). Through this 3-way valve, 40 ml of medium was flushed into the uterus via the Fallopian tubes using a 20 ml syringe to ensure that all tubal stage embryos were accessed via flushing and collected. An embryo flushing catheter was previously inserted into the respective uterine horns, as is routinely done for embryo retrieval on Day 7. One end of the flushing catheter was connected to an EmCon filter in which the embryos were collected (Havlicek et al., 2018).

### Combining transfer and re-collection for in vivo culture

An initial attempt was made to transfer embryos into the oviducts of heifers. Oviduct access was achieved in all heifers, with 23 transfers ipsilateral and 1 transfer contralateral. A total of 26 in vitro derived 2- to 4-cell stage embryos were transferred, 9 animals were found to be pregnant and 7 calves were born (Besenfelder and Brem, 1998). The next step was to flush the embryos out of the Fallopian tubes. This was achieved step by step. In one group, animals were inseminated following heat detection, while another group underwent superovulation treatment. The superovulated animals were additionally divided into two groups. As this is the first time this technology has been tested, only one side of the first hormone treated group was flushed, i.e. one oviduct and the corresponding uterine horn. In the second group, both sides were included in the embryo collection. Here, too, it was impressively demonstrated that almost all tubal stage embryos from all groups of animals could be retrieved using this flushing method (Besenfelder et al., 2001).

Consequently, and in the final step, the transfer of the in vitro derived embryos and their re-collection were combined for in vivo culture, i.e. two endoscopic procedures were performed. The time interval between the two procedures corresponded to the duration of the in vivo culture of the embryos. In general, it has been shown that in vitro generated embryos can be cultured in bovine oviducts and that their developmental capacity is close to that of embryos obtained from cows after superovulation.

However, it has also been shown that the in vitro maturation process results in an oocyte that is not suitable for transfer into the oviduct of previously inseminated heifers. The results of oocyte maturation in the oviduct are still too different from those obtained in the laboratory. The sperm in the oviduct obviously do not manage to successfully fertilize the in vitro collected oocyte (Wetscher et al., 2005a).

In addition, the physiological process of ovulation produces an expanded cumulus-oocyte complex with special adhesion properties within a viscous follicular fluid, which must be taken into account when transferring embryos previously generated in vitro to ensure development and correct migration of the embryos in the oviduct. In addition, the time of transfer after ovulation plays a crucial role in relation to the ciliary and muscular activity of the Fallopian tube (Wetscher et al., 2005b).

### Scientific approaches to assess the developmental competence of the embryo

Once the above steps had been developed and established, it was possible to analyze early embryonic development for various environmental influences using numerous scientific approaches. The aim was then to use these findings to pursue strategies that would have a lasting impact on embryonic development both, in vitro as well as in vivo. The following are examples of studies in which this influence has been demonstrated.

A closer look at the Zona pellucida provides an example of the diverse and active nutritional situation of the embryo in the oviduct. The Zona pellucida represents a filter system, that also separates the embryonic cells from the oviductal epithelium and fluid. Active molecular exchange processes are clearly reflected in the dynamic structure of the zona. It has been shown that the zona increases in thickness in embryos gained from the oviduct and that the reticular structure is increasingly covered by vesicles from the oviduct. Moreover, the structure of the Zona pellucida of in vitro embryos indicates some degeneration of the outer layers. Whereas, in vivo the cilia at the border of the embryonic cells to the Zona pellucida are stronger and denser, and the zona of the embryos exhibits greater elasticity after culture in the oviduct (Mertens et al., 2006, 2007). This active molecule exchange is also reflected in the membrane stability to cryo-influences on the embryo with increasing residence time in the oviduct (Havlicek et al., 2010).

### Early embryonic stages in cattle: critical developmental points and environmental influences

In a large-scale study, embryos were removed from the oviduct at various times and cultured in vitro to the blastocyst stage to investigate the developmental steps in the early embryonic phase. Conversely, embryos from the in vitro preparation were transferred into the oviduct at different times and removed from the animal at the blastocyst stage. All blastocysts were compared by expression analysis with blastocysts that had developed exclusively in the animal. In general, it was found that the number of developing blastocysts was dependent on the in vitro or in vivo origin, i.e. significantly more oocytes/embryos whose development start was in vivo reached the blastocyst stage compared to the in vitro oocytes/embryos whose development start was in vitro. Overall, this study also showed that the fertilization process, the 8-cell stage where genome activation takes place, and the compaction process are particularly the most critical steps performed in vitro (Gad et al., 2012). This appearance of the in vitro generated embryos could also be confirmed by epigenetic studies (Salilew-Wondim et al., 2015, 2018).

Other studies have also shown that in vitro culture conditions contribute to an alarming embryo maldevelopment. This was demonstrated by collecting COCs for IVP and by flushing early tubal stage embryos from in the same donor animals. Individual blastomeres were isolated from early-stage embryos, the entire genome was amplified and finally hybridized to an Illumina BovineHD BeadChip array. The frequency and nature of chromosomal instability (CIN) differs significantly between in vivo and in vitro cultured cattle. In in vitro methods, whole chromosome and segmental abnormalities increase significantly during early embryonic development (Tšuiko et al., 2017).

Hormonal treatment of donor animals to obtain early embryonic stages (MOET programs) also showed clear differences depending on whether they were removed early from the treated donor animals and transferred to single-ovulated animals or remained in the hormonally treated animal until blastocyst. Studies of gene expression and developmental dynamics clearly illustrate that hormone treatment of donor animals causes environmental changes that negatively affect these early embryonic stages (Gad et al., 2011).

Following the influences on early embryonic development already described, it is easy to see that lactation in cows also might have a significant influence on these stages. Embryos generated in vitro at an early stage were transferred into the oviducts of heifers (with/without progesterone supplementation), heifers or lactating cows, or post-partum cows (lactating or dried off) and recovered. These experiments have clearly shown that both reduced blood progesterone levels and milk production have a detrimental effect on embryonic development, i.e. cows with high milk yield do not have sufficient metabolic capacity to additionally support the development of the embryo (Carter et al., 2010; Rizos et al., 2010; Maillo et al., 2012).

Finally, it should be emphasized that the use of endoscopy for embryo collection and transfer offers the unique advantage of being able to determine the cycle status or the age of the corpus luteum very accurately before, at the time of expected ovulation and hours to days after ovulation. In this way, over the years, the various treatment protocols have been continuously optimized or adapted to the respective husbandry situation in order to achieve optimal synchronization of the animals. It should be noted that synchronization is a very important starting point for any study of embryonic development. Three essential areas are highlighted, all of which need to be combined in the context of synchronization:

- Individual animal level: the animal should be in a defined ovarian cycle
- Group level: all animals should be in the same desired synchronized state
- Follicular wave: the follicular dynamics should also correspond to the cycle state, i.e. be synchronized.

A specially designed and conducted study has clearly highlighted that synchronization is very important in cattle. Deviations can have serious negative effects on embryo development (Rodríguez-Alonso et al., 2020).

## Current approaches looking to the future

Many important novel questions have arisen from the previous studies and their results, which are already being addressed or will be addressed by our group in the near future. For example, we were able to show that sperm of different origin and in some cases in very small quantities can be released directly into the oviduct, where successful fertilizations can take place (Radefeld et al., 2018). As oocytes and embryos can be tracked and identified culturally, it would be interesting in the future to be able to record the smallest amounts of sperm and oocytes as a whole in the Fallopian tube in order to more accurately record and thus define other fertility parameters.

Following the development of embryo transfer, its flushing and in vivo culture, the current focus is on the collection of oviduct fluid to determine total protein content (Papp et al., 2019) and even more so on the collection of fluid at a defined time after ovulation and insemination, including the corresponding embryo (Havlicek et al., 2022). This would provide key components for in vitro culture and would increase information on the factors that determine fertility. In addition, these embryos are an important tool for implementing valuable breeding techniques (Miskel et al., 2019).

Last but not least, more emphasis should be placed on clinical applications in the future, focusing on post-pubertal events and the influence of heat stress (as the sum of climatic and metabolic stress), especially the development of endometritis (Neubrand et al., 2021; Pothmann et al., 2022).

## Conclusion

As it can be seen from this report, the focus of our work has been on the inclusion of the Fallopian tube in biotechnological measures and on the investigation of fertility in the early embryonic stage. In the future, a major area of application will be the ever-increasing number of IVPs worldwide, which will inevitably stimulate, if not necessitate, improvements in in vitro culture. On the one hand, a plethora of important factors for in vitro culture can be identified. On the other hand, the direct use of the potential of the oviduct for healthy embryo development represents the provision of all cellular and molecular factors in chronological order. It is believed that the combination of science and practice described here is very much in the spirit of the Association for Embryo Technology in Europe, AETE.

As we are the only team in the world now routinely using this technique, we hereby invite all ET teams, especially those working with IVP-derived embryos, to give more importance to the oviduct in the development of early embryonic stages, both for breeding purposes and for further scientific applications.

## Data Availability

Research data is available in the body of the article.
